# Comprehensive competitive endogenous RNA network analysis reveals *EZH2*-related axes and prognostic biomarkers in hepatocellular carcinoma

**DOI:** 10.22038/IJBMS.2022.61570.13623

**Published:** 2022-03

**Authors:** Mohammad Hossein Donyavi, Sadra Salehi-Mazandarani, Parvaneh Nikpour

**Affiliations:** 1 Department of Cell and Molecular Biology and Microbiology, Faculty of Biological Science and Technology, University of Isfahan, Isfahan, Iran; 2 Department of Genetics and Molecular Biology, Faculty of Medicine, Isfahan University of Medical Sciences, Isfahan, Iran; 3 Child Growth and Development Research Center, Research Institute for Primordial Prevention of Non-communicable Disease, Isfahan, Iran

**Keywords:** Biomarkers, Carcinoma, Competing- endogenous - RNA, Circular RNA, Hepatocellular, Long Noncoding RNA

## Abstract

**Objective(s):**

Hepatocellular carcinoma (HCC) is a common and lethal type of cancer worldwide. The importance of non-coding RNAs such as long non-coding RNAs (lncRNAs), circular RNAs (circRNAs), and microRNAs (miRNAs) have been recognized in the development of HCC. In this study, we constructed a four-component competing endogenous RNA (ceRNA) network in HCC and evaluated prognostic values of the ceRNAs.

**Materials and Methods:**

The expression profiles of lncRNAs, miRNAs, and mRNAs were retrieved from The Cancer Genome Atlas database. GSE94508 and GSE97332 studies from the Gene Expression Omnibus database were used to identify circRNAs expression profiles. A four-component ceRNA network was constructed based on differentially-expressed RNAs. Survival R package was utilized to identify potential prognostic biomarkers.

**Results:**

A four-component ceRNA network including 295 edges and 239 nodes was constructed and enrichment analysis revealed important Gene Ontology and Kyoto Encyclopedia of Genes and Genomes pathways. A Protein-Protein Interaction network with 118 nodes and 301 edges was also established. The enhancer of zeste homolog 2 (EZH2) was the highest degree hub gene in the PPI network. Because of the significance of EZH2 in HCC, we presented its axes in the ceRNA network, which play important roles in HCC progression. Furthermore, ceRNAs were identified as potential prognostic biomarkers utilizing survival analysis.

**Conclusion:**

Our study elucidates the role of ceRNAs and their regulatory interactions in the pathogenesis of HCC and identifies EZH2-related RNAs which may be utilized as novel therapeutic targets and prognostic biomarkers in the future.

## Introduction

Hepatocellular carcinoma (HCC) is the most common type of liver cancer, accounting for approximately 80% of cases and ranking fourth cause of cancer-related deaths worldwide (1-3). HCC has a poor prognosis because it is usually detected at the advanced stages. There are only a few therapeutic options with limited health benefits for the treatment of HCC (4, 5). Therefore, new prognostic and diagnostic biomarkers and novel therapeutic targets are required.

Different types of RNAs have been shown to compete for binding to the same microRNAs (miRNAs) through their miRNA response elements (MREs). These interactions and competitions among RNAs referred to as competing endogenous RNAs (ceRNAs) result in the formation of ceRNA networks (6). The investigation of these networks will contribute to a better understanding of the molecular mechanisms underlying cancer progression as well as the development of new biomarkers and therapeutic targets.

ceRNA networks are important for HCC progression (7). The roles of lncRNAs and circRNAs as ceRNAs in miRNA-mediated gene expression modulation in HCC have been demonstrated over the last decade (8, 9). Xiong Dd *et al. *established a circRNA-miRNA-mRNA ceRNA network in HCC and identified 7 hub genes involving in cancer-related pathways and biological functions (10). Luo Y *et al. *constructed a lncRNA-miRNA-mRNA ceRNA network in HCC and identified four genes associating with survival (*CCNA2*, *CHEK1*, *FOXM1 *and *MCM2*) (11). Another study established a ceRNA network in HCC and identified 9 lncRNAs as hub genes enriched in various cancer-related processes (12).

Although there are several studies demonstrating the vital interactions and functions of ceRNAs in HCC, little is known about the four-component ceRNA network in which lncRNAs, circRNAs, and mRNAs compete for binding to the same miRNAs. These highly informative networks, which include four distinct types of ceRNAs, can provide a more comprehensive picture of the regulatory interactions occurring in cancer.

**Figure 1:**
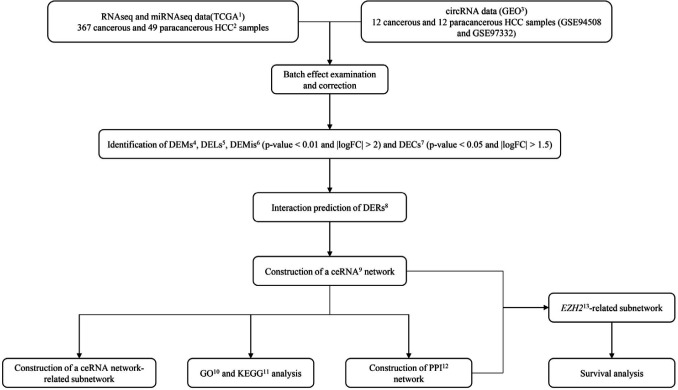
The main flow-chart of this study. 1- The Cancer Genome Atlas, 2- Hepatocellular carcinoma, 3- Gene Expression Omnibus, 4- Differentially expressed mRNAs, 5- Differentially expressed lncRNAs, 6- Differentially expressed miRNAs, 7- Differentially expressed circRNAs, 8- Differentially expressed RNAs, 9- Competing endogenous RNA, 10- Gene ontology, 11- Kyoto encyclopedia of genes and genomes, 12- Protein-protein interaction, 13- Enhancer of zeste homolog 2

Using the Cancer Genome Atlas (TCGA) and the Gene Expression Omnibus (GEO) databases, we identified differentially expressed lncRNAs, miRNAs, mRNAs, and circRNAs in cancerous versus paracancerous HCC tissues (13, 14). Their interactions were then analyzed, and a ceRNA network comprised of all four types of mentioned RNAs was constructed. We used mRNAs of the ceRNA network to identify Gene Ontology (GO) and Kyoto Encyclopedia of Genes and Genomes (KEGG) pathways and to construct a Protein-Protein Interaction (PPI) network. Furthermore, survival analysis was performed to identify ceRNAs which may serve as prognostic biomarkers in HCC. The pipeline of the current study is depicted in Figure 1.

## Materials and Methods

***RNA sequencing data***

A total of 422 HCC miRNA-seq data samples (372 cancerous and 50 paracancerous) and 421 HCC RNA-seq data samples (371 cancerous and 50 paracancerous) were retrieved from the TCGA database using the TCGAbiolinks package in the RStudio software (15). Then, samples from patients with both RNA-seq and miRNA-seq data (367 cancerous and 50 paracancerous data samples for each RNA-seq and miRNA-seq data) were selected for further analysis. 

***Batch effect examination and correction***

The batch effect is a complication that researchers face when analyzing high-throughput data with technical differences and results from non-biological conditions that affect the results of studies (16, 17). The swamp package in the RStudio software was used to identify strong batch effects in RNA-seq data (containing Tissue Source Site (TSS), portion, and plate) and miRNA-seq data (containing TSS, portion, and plate). Then, batch effect correction was performed on both RNA-seq and miRNA-seq data by using ComBat_seq from the sva package (18).

***Microarray data***

The raw gene expression profiles of circRNAs in HCC were retrieved from the GEO database (https://www.ncbi.nlm.nih.gov/geo/) consisting of GSE94508 (including 5 cancerous and 5 paracancerous samples) and GSE97332 (including 7 cancerous and 7 paracancerous samples). The two datasets were combined, and the batch effect was removed through the sva package (19).

***Identification of differentially expressed RNAs (DERs)***

We compared 367 cancerous and 49 paracancerous RNA-seq and miRNA-seq data samples in order to identify differentially expressed mRNAs (DEMs), lncRNAs (DELs), and miRNAs (DEMis), using the DESeq2 package, with the threshold of |log2FC| > 2 and adjusted *P*-value < 0.01 (20). To investigate differentially expressed circRNAs (DECs) in 12 cancerous and 12 paracancerous samples, the limma R package was utilized with the threshold of |log2FC| > 1.5 and adjusted *P*-value < 0.05 (21). The ggplot2 and pheatmap packages were utilized to generate volcano and heatmap plots of DERs in the RStudio software.

***Interaction prediction and construction of a ceRNA network***

We annotated RNA-seq data using the biomaRt R package based on the Ensembl genome browser (22). We utilized the multiMiR R package to retrieve interactions between DEMs and DEMis (23). Three databases, including the TargetScan, miRDB, and miRTarBase, were selected via the multiMiR package for this aim and 20% of the most reliable interactions were retrieved (24-26). The interactions with a threshold of Context++ score ≤ - 0.6 were obtained from the TargetScan database. Additionally, only interactions with strong evidence were selected from the miRTarBase database. DELs and DEMis interactions were retrieved from the RNAInter online database with a score > 0.5 (27). We used the CircInteractome online database to identify DECs and DEMis interactions with context+ score percentile ≥ 90 (28). The ceRNA network was constructed using Cytoscape software (version 3.8.1) (29). A sub-network was established by using “cytoHubba” (a plugin in Cytoscape software) for identification of hub genes in the network based on their topological feature (degree) (30).

***Functional enrichment analysis***

The KOBAS 3.0 online web tool was used to identify GO and KEGG pathways relating to the ceRNA networkʼs DEMs (31). The corrected *P*-value < 0.001 was considered as a significant cut-off for GO analysis. Moreover, the corrected *P*-value<0.01 was considered as a significant cutoff for KEGG analysis. The ggplot2 R package and Cytoscape software were used to visualize KEGG pathways and the genes associated with them. Furthermore, the GOplot R package was utilized to visualize the results of the GO analysis (32).

***Establishment of a PPI Network and a sub-network***

We established a PPI network based on the ceRNA networkʼ DEMs, using the online STRING database (33). Subsequently, in Cytoscape, free nodes were eliminated, and hub genes were detected via cytoHubba. The first hub gene (according to the degree feature) was selected to construct a sub-network based on its interactions from the ceRNA network.

***Survival analysis***

Survival analysis was performed on the ceRNA sub-networkʼs DEMs, DEMis, and DELs to explore potential HCC prognostic biomarkers. The survival R package was utilized for survival analysis. Cancerous samples were divided into low and high expression groups based on the mean expression of each RNA, and the Kaplan-Meier method was employed to perform the analysis. *P*-value<0.05 and Hazard Ratio (HR) ≠ 1 were considered as statistically significant thresholds. The clinical information of the patients were extracted from the TCGA database using the TCGAbiolinks R package. The survminer package was utilized for drawing survival plots. Box plots were also generated using ggpubr R package.

***Statistical analyses***

Spearman correlation among miRNA-seq and RNA-seq samples were performed separately using the TCGAbiolinks R package based on correlation > 0.7. The survivalROC package was utilized to examine prognostic values using the receiver operation curve (ROC) plot (34).

## Results

***Batch effects examination and correction in TCGA data***

There were 367 cancerous and 50 paracancerous miRNA-seq and RNA-seq data samples from the same HCC patients. The swamp R package was used to generate prince plots to investigate batch effects in RNA-seq and miRNA-seq data. Both miRNA-seq and RNA-seq data have been shown to exhibit significant batch effects based on their plate ID (Supplementary Figure 1a and b). Due to the statistical limitations of ComBat_seq, data from a paracancerous sample with a unique batch ID were omitted from miRNA-seq and RNA-seq data. Finally, 367 cancerous and 49 paracancerous data samples remained for RNA-seq and miRNA-seq data analyses, respectively. The batch effect was then corrected according to plate ID in both RNA-seq and miRNA-seq data (Supplementary Figure 1c and d).

***Differentially expressed RNAs in HCC***

**Figure 2.**
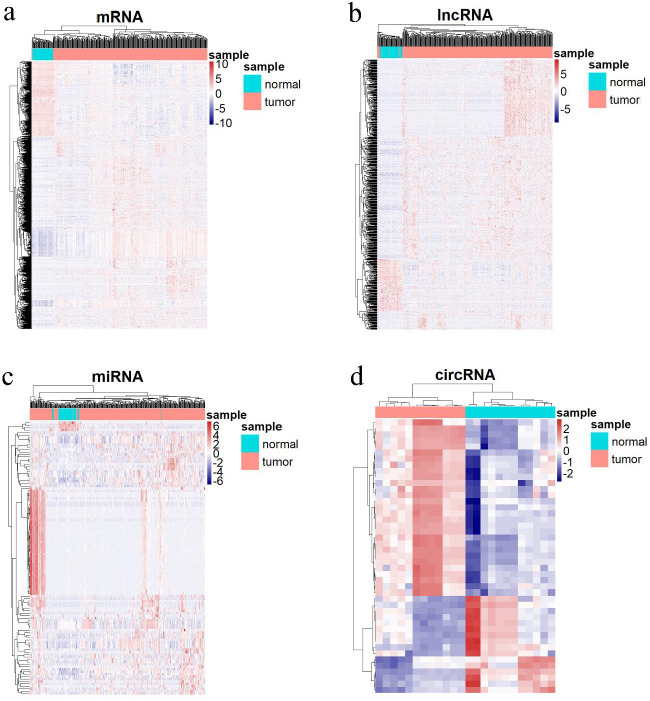
The heatmap plots show detailed expression patterns of differentially expressed RNAs between cancerous and paracancerous HCC samples. a) Heatmap plot of DEMs, b) Heatmap plot of DELs, c) Heatmap plot of DEMis, d) Heatmap plot of DECs. Blue and red colors represent low and high expression of RNAs, respectively. The horizontal and vertical axes represent samples and differentially expressed RNAs, respectively

**Figure 3.**
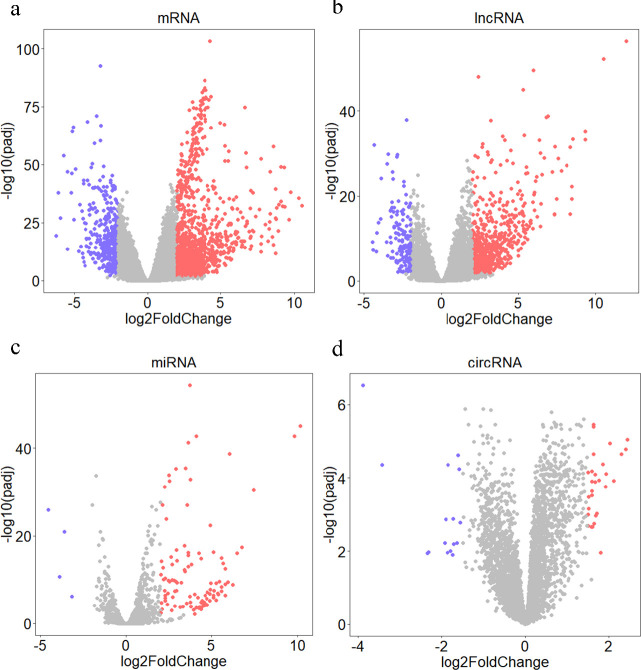
The volcano plots show differentially expressed RNAs between cancerous and paracancerous HCC samples. a) Volcano plot of DEMs, b) Volcano plot of DELs, c) Volcano plot of DEMis. |log2FC|>2 and adjusted *P*-value<0.01 are considered as significant thresholds for DEMs, DELs, and DEMis. d) Volcano plot of DECs. |log2FC| >1.5 and adjusted *P*-value<0.05 are considered as significant thresholds for DECs. Blue and red colors represent low and high expression of RNAs, respectively. The horizontal and vertical axes represent -log2 (fold change) and -log10 (adjusted *P*-value)

Based on the threshold of |log2FC| > 2 and adjusted *P*-value<0.01, 755 DELs (608 upregulated and 147 downregulated), 1509 DEMs (1089 upregulated and 420 downregulated) and 113 DEMis (109 upregulated and 4 downregulated) were identified in HCC. Additionally, 45 DECs (29 upregulated and 16 downregulated circRNAs) were identified based on the threshold of |log2FC| > 1.5 and adjusted *P*-value<0.05 in cancerous compared to paracancerous HCC samples. The volcano and heatmap plots depict DERs and the distribution of their expression in cancerous and paracancerous HCC samples (Figure 2 and 3).

***Interaction prediction and constructing a ceRNA network***

Utilizing the TargetScan, miRTarBase, and miRDB databases via the multiMiR R package, 1078 interactions existing at least in one of the three mentioned databases were identified between DEMs and DEMis containing 95 unique DEMis. Among the 95 DEMis interacting with DEMs, CircInteractome and RNAInter databases revealed 24 DECs-DEMis and 8 DELs-DEMis interactions, respectively. Collectively, 297 interactions were identified and used to construct a ceRNA network. Then, a four-component ceRNA network was constructed with 239 nodes and 295 edges (one axis which had no connection to the other parts of the network was deleted (*ASB15*/ hsa-miR-1251-5p/ hsa_circ_0072088).

**Figure 4:**
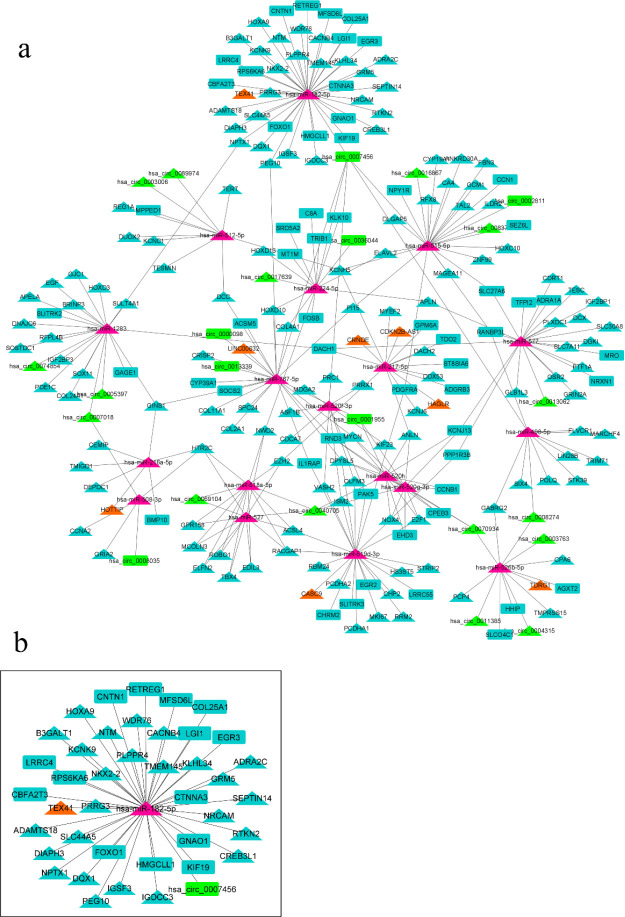
ceRNA network and a sub-network in HCC. a) ceRNA network including DELs (orange), DECs (green), DEMs (blue) and DEMis (purple). b) A sub-network was extracted from the ceRNA network. hsa-miR-182-5p has the highest degree in the ceRNA network. Triangle and rectangle shapes represent upregulated and downregulated RNAs, respectively

The four-component ceRNA network included 8 lncRNAs (8 upregulated), 23 circRNAs (16 upregulated and 7 downregulated), 18 miRNAs (18 upregulated), and 190 mRNAs (133 upregulated and 57 downregulated) (Figure 4a). According to the network’s topological feature (degree), hsa-miR-182-5p had the highest degree (degree = 41) and was selected for extracting its sub-network from the ceRNA network (Figure 4b). Moreover, hsa_circ_0007456 was a hub gene in the ceRNA network (it was the first hub gene based on the degree feature (degree = 6) of all nodes except miRNAs). Interestingly, hsa-miR-182-5p interacted with hsa_circ _0007456 in the ceRNA network.

***Functional enrichment analysis***

**Figure 5:**
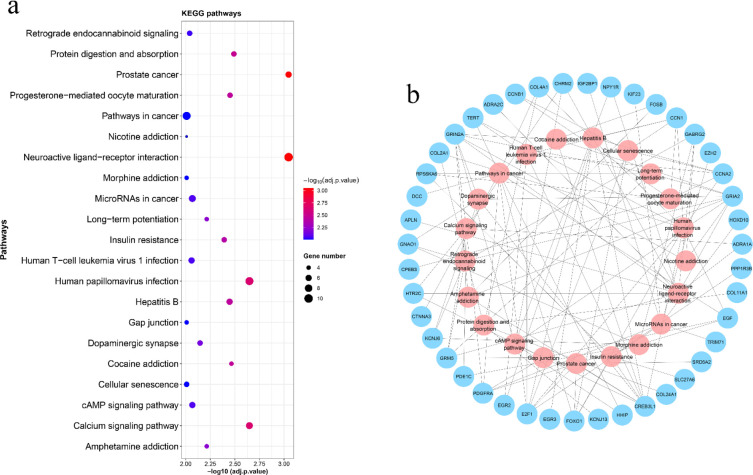
Significant KEGG pathways relating to the DEMs of the ceRNA network. a) A dot plot showing significant KEGG pathways. The Y axis represent the name of each signaling pathway and X axis represent -log10 (adjusted *P*-value) of each pathway. The size of the dots indicates the number of genes involved in the related signaling pathway. The significance level of the pathways is shown by changes in the color scale. b) A network including signaling pathways and the genes relating to them. Pink and blue circles represent pathways and genes, respectively

**Figure 6:**
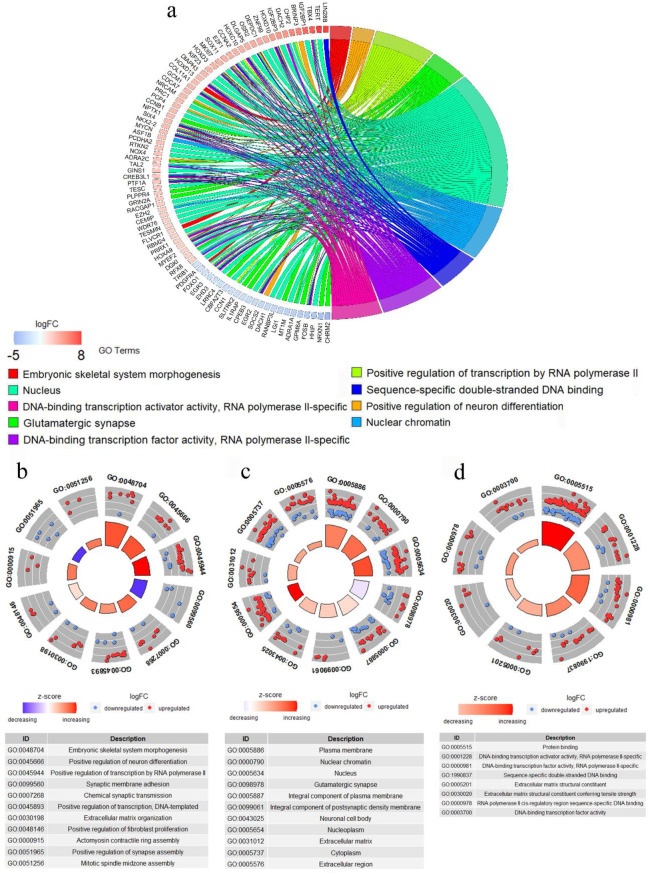
GO analysis based on the DEMs of the ceRNA network. a) The GOChord plot of the most significant three gene ontology terms relating to each GO groups (biological process, cellular component, and molecular function). b) GOCircle plot of biological process part of GO analysis. c) GOCircle plot of cellular component part of GO analysis. d) GOCircle plot related to molecular function part of GO analysis

KEGG pathway analysis showed significant pathways such as calcium signaling pathway, protein digestion and absorption, hepatitis B, microRNAs in cancer, gap junction, pathways in cancer, and cellular senescence with corrected *P*-value < 0.01 (Figure 5). On the other hand, GO enrichment analysis revealed 30 terms with corrected *P*-value < 0.001 (Figure 6). Among these GO terms, biological process (BP), cellular component (CC), and molecular function (MF) were associated with 11, 11, and 8 terms, respectively. The supplementary files contain all significant KEGG pathways (corrected *P*-value < 0.01) and GO terms (corrected *P*-value < 0.001) (Supplementary file 1 and 2).

***Protein-protein interaction (PPI) and screening of hub genes***

**Figure 7:**
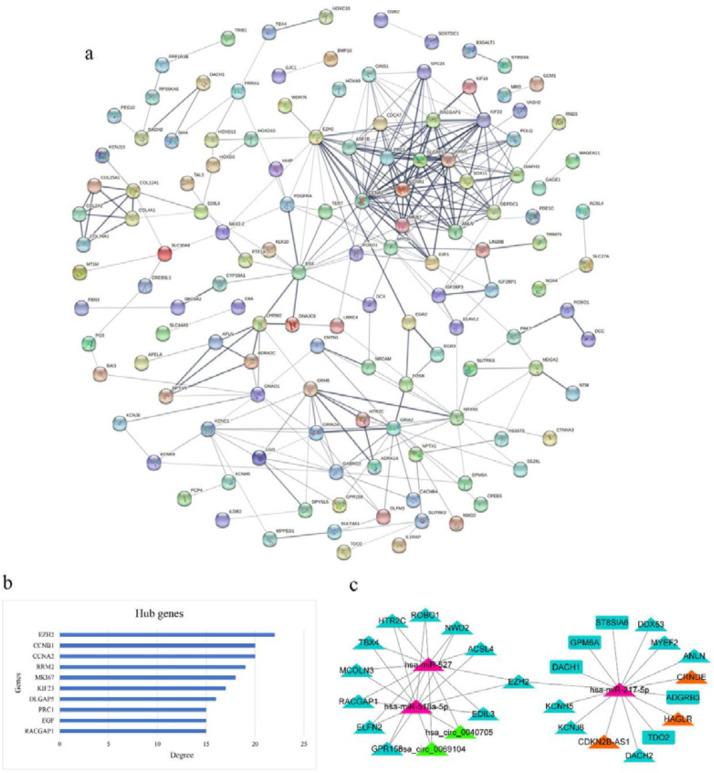
The protein-protein interaction (PPI) network constructed based on the mRNAs from the ceRNA network and its degree-based examination. a) The PPI network. b) Hub genes of the PPI network based on degree feature. C) *EZH2*-related sub-network including DELs (orange), DECs (green), DEMs (blue) and DEMis (purple). Triangle and rectangle shapes represent upregulated and downregulated RNAs, respectively

The ceRNA network-related mRNAs were utilized for constructing a PPI network. A PPI network with 118 nodes and 301 edges was generated using the STRING online database (Figure 7a). There were 10 hub genes in the network with degree greater than 15, which the enhancer of zeste homolog 2 (EZH2) had the highest degree (Figure 7b). EZH2 plays a critical role in HCC (35, 36). As a result, we investigated its ceRNA network axes and introduced both lncRNAs and circRNAs as competitors for *EZH2*. *EZH2* was found to be associated with 7 axes which correlated with circRNAs or lncRNAs (hsa_circ_0040705-hsa-miR-527-*EZH2* / hsa_circ_0040705-hsa-miR-518a-5p–*EZH2* / hsa_circ_0069104-hsa-miR-527-*EZH2* / hsa_circ_0069104-hsa-miR-518a-5p–*EZH2 */ *HAGLR*-hsa-miR-217-5p–*EZH2* / *CRNDE*-hsa-miR-217-5p–*EZH2*/*CDKN2B-AS1*–hsa-miR-217-5p–*EZH2)*. Subsequently, the *EZH2* sub-network was established (Figure 7c).

***Survival-related RNAs***

**Figure 8:**
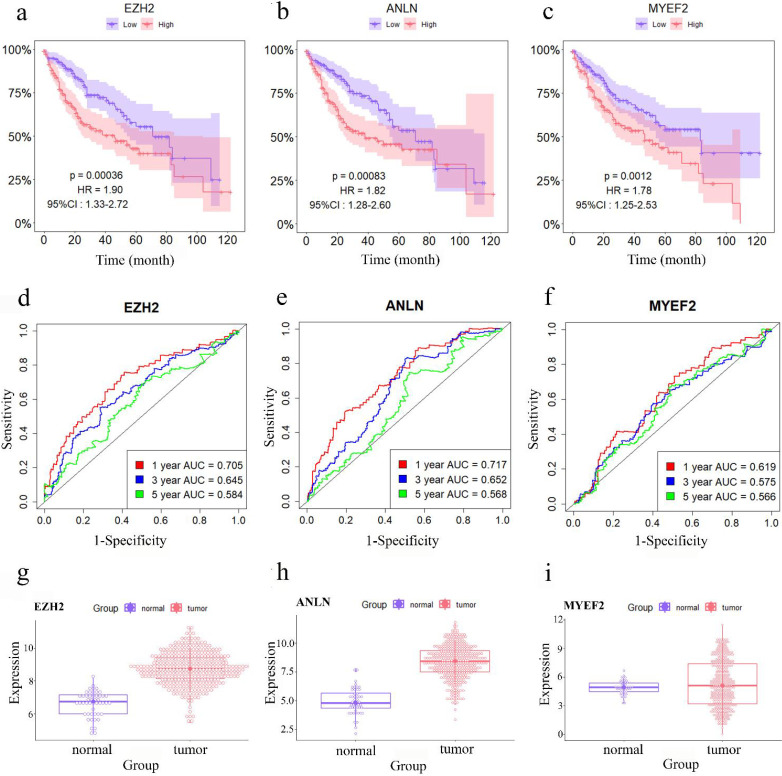
Survival-related and box plots of the three survival-related ceRNAs. a) Survival plot of *EZH2*, b) Survival plot of *ANLN*, c) Survival plot of *MYEF2*. Purple and pink colors represent low and high gene expression in samples. Y and X axes represent survival percentage of patients and time, respectively. d) Receiver Operating Characteristic (ROC) curve plot of *EZH2*, e) ROC curve plot of *ANLN*, f) ROC curve plot of *MYEF2*. Red, blue and green colors represent one, three and five-year survival area under the ROC curve (AUC), respectively. g) Boxplot of *EZH2*, h) Boxplot of *ANLN*, i) Boxplot of *MYEF2*. Purple and pink colors represent paracancerous and cancerous HCC samples, respectively

Univariate survival analysis was conducted to identify survival-related RNAs from the sub-network of *EZH2*. A total of 13 RNAs (10 mRNAs, 2 lncRNAs, and 1 miRNA) were revealed as survival-associated RNAs in HCC based on *P*-value<0.05 and HR ≠ 1 (Table 1). Among these DERs, *EZH2*, *ANLN*, and *MYEF2* had the highest HR and HCC patients who expressed higher levels of these RNAs had poorer survival rate (Figure 8a-f). Roc curve plots indicated that these three RNAs could be effective prognostic biomarkers in HCC patients (Figure 8d-f). The expression changes of these three RNAs between cancerous and paracancerous samples have also been shown (Figure 8g-i).

## Discussion

HCC is the fourth cause of cancer-related death in the world (1). While numerous studies have been conducted to elucidate the molecular mechanisms underlying the progression of HCC, they have not been completely understood. lncRNAs and circRNAs are two types of ceRNAs which their competition affects the development of cancers, including HCC (8, 9). Identification of these interactions will aid in our understanding of the molecular mechanisms underlying HCC and the development of novel diagnostic and prognostic biomarkers.

The current study investigated the interactions among four types of RNAs in HCC, including lncRNAs, circRNAs, mRNAs, and miRNAs and their four-component ceRNA network. After correcting batch effects, we identified 755 DELs, 1509 DEMs, 113 DEMis, and 45 DECs between cancerous and paracancerous HCC samples. A ceRNA network with 239 nodes and 295 edges was constructed based on the predicted interactions of DERs. Enrichment analysis was then performed based on the mRNAs from the network and revealed significant signaling pathways and GO terms. These results can help us to better understand molecular and cellular processes involving in the development of HCC. The mRNAs from the ceRNA network were then utilized to establish a PPI network. EZH2 had the highest degree among the hub genes in the PPI network.

Examining ceRNAs from the ceRNA network revealed hsa-miR-182-5p as the highest degree hub gene and hsa_circ_0007456 as a hub gene based on degree of all nodes except miRNAs. The two hub genes directly interact with each other in the ceRNA network, and their sub-network was extracted. miR-182-5p induces proliferation of HCC cells through AKT/FOXO3a signaling pathway, and its increased expression is associated with poor prognosis in HCC patients (37). Downregulation of hsa_circ_0007456 in HCC tissues has been reported and it can affect natural killer cell-mediated cytotoxicity through the hsa_circ_0007456/miR-6852-3p/ICAM-1 axis in HCC (38).

EZH2, a critical transcription regulator, is a subunit of the poly comb repressive complex 2 (PRC2), which can influence the expression of its target genes in various ways, such as methylation of Lys-27 in histone 3 (39, 40). Overexpression of EZH2 has been identified in various cancers, including gastric cancer, thyroid carcinoma, prostate cancer, and HCC (36, 41-43).

EZH2 overexpression is associated with HCC progression and metastasis (35). EZH2 can induce tumorigenesis by affecting tumor-suppressive genes and initiating cancer-related molecular processes like miRNA silencing, non-canonical transcription regulation, and NF-kB activation (44). Therefore, new strategies for manipulation of EZH2 may be developed to improve cancer therapy in the near future (44). 

Different studies have identified the role of EZH2 as an epigenetic modifier in HCC. In a study conducted by Au SL *et al*. it was revealed that EZH2 can epigenetically inhibit the expression of tumor-suppressor miRNAs, such as miR-139-5p, miR-125b, miR-101, let-7c, and miR-200b in HCC (35). Another study demonstrated that the expression of immune checkpoint inhibitor, programmed death-1 ligand 1 (PD-L1), can be suppressed by EZH2 in HCC through methylation of its encoding gene and IRF1 which is an essential transcription factor for the expression of PD-L1 (45). Xu L *et al.* revealed that ectopic overexpression of miR-101 as a tumor suppressor inhibits the progression, invasion, and proliferation of HCC by directly targeting and decreasing the expression of *EZH2* (46). A study published in 2019 constructed a ceRNA network in HCC and demonstrated the interaction of *EZH2* and its competitors with hsa-mir-217. Some of its competitors are also identified in this study such as *DACH1* and *CRNDE* (47).

In the *EZH2* related sub-network, three miRNAs (hsa-miR-527, hsa-miR-518a-5p, and hsa-miR-217-5p) interacted with *EZH2*. It has been reported that miR-527 may be involved in the progression of HCC by targeting Glypican-3 (48). Contribution of miR-217 in tumor progression of several cancer types including hepatocellular carcinoma has been documented (49).

In the present study, we demonstrated that overexpression of *EZH2* was negatively associated with overall survival in HCC patients, with the highest hazard ratio of all survival-related RNAs. Previous studies have established that overexpression of EZH2 is associated with a poor prognosis in various types of cancer (50). Notably, it has been reported that overexpression of EZH2 is associated with tumor progression, aggressiveness, and poor prognosis in HCC (45, 51-54). A recent study conducted by Zhang *et al*. revealed that EZH2 is overexpressed in HCC and correlates with poor survival. They indicated that silencing EZH2 inhibits the viability, migration, and invasion of HCC cells via the TGF-β-MTA1-SMAD7-SMAD3-SOX4-EZH2 signaling cascade (52). Additionally, Guo *et al.* reported that high EZH2 expression is associated with poor overall survival, disease-specific survival, progression-free survival, and relapse-free survival in nearly all patients with HCC. Furthermore, they discovered a relationship between EZH2 and major MHC class I antigen presentation molecules, indicating that it plays an immunosuppressive role (53). In another study, Xu *et al*. revealed that increased EZH2 expression is associated with increased tumor size, metastasis, relapse, and an unfavorable prognosis in HCC (54). Collectively, these findings suggest that EZH2 may be a useful prognostic biomarker in patients with HCC. 

**Table 1:** Survival-related ceRNAs from the sub-network of EZH2. The columns of group and patients represent the number of patients with high and low expression of each RNAs. †: hazard ratio

Type	Name	Group	Patients	P-value	HR ^†^
	*EZH2*	High Low	185 181	0.00036	1.9
	*ANLN*	High Low	187 179	0.00081	1.82
	*MYEF2*	High Low	174 192	0.0012	1.78
	*TBX4*	High Low	130 236	0.0017	1.74
mRNA	*MCOLN3*	High Low	149 217	0.0022	1.72
	*ROBO1*	High Low	214 152	0.0064	1.66
	*RACGAP1*	High Low	191 175	0.0076	1.62
	*KCNJ6*	High Low	144 222	0.028	1.47
	*DDX53*	High Low	124 242	0.034	1.47
	*GPM6A*	High Low	187 179	0.015	0.65
	*CDKN2B-AS1*	High Low	198 168	0.005	1.67
lncRNA	*HAGLR*	High Low	177 189	0.016	1.54
miRNA	hsa-miR-217-5p	High Low	186 179	0.016	1.55

## Conclusion

Our study identifies a novel four-component ceRNA network consisting of four types of RNAs in HCC and reveals *EZH2*-related ceRNA axes. Future studies will elucidate the precise roles of these axes in the pathogenesis of HCC. Three miRNAs (hsa-miR-527, hsa-miR-518a-5p, and hsa-miR-217-5p) interacting with *EZH2*, may be used to inhibit *EZH2*ʼs oncogenic function in the future. Furthermore, we identified ceRNAs associated with survival in the sub-network of *EZH2*, which may be helpful in survival-related research aimed at identifying prognostic biomarkers in HCC. Further studies are needed to examine and validate these conclusions experimentally.

## Ethical Approval

Approval was obtained from the ethics committee of Isfahan University of Medical Sciences, Isfahan, Iran. The procedures used in this study adhere to the tenets of the Declaration of Helsinki.

## Authors’ Contributions

PN Designed the study; MHD and SSM Collected data and performed analyses; PN, SSM and MHD Discussed the results and strategy; PN Supervised, directed and managed the study; MHD, SSM and PN Drafted the manuscript and approved the final version to be published.

## Conflicts of Interest

The authors report no conflict of interest.
